# The Fate and Distribution of Autologous Bone Marrow Mesenchymal Stem Cells with Intra-Arterial Infusion in Osteonecrosis of the Femoral Head in Dogs

**DOI:** 10.1155/2016/8616143

**Published:** 2015-12-08

**Authors:** Hongting Jin, Taotao Xu, Qiqing Chen, Chengliang Wu, Pinger Wang, Qiang Mao, Shanxing Zhang, Jiayi Shen, Peijian Tong

**Affiliations:** ^1^Zhejiang Chinese Medical University, Hangzhou, Zhejiang 310053, China; ^2^Institute of Orthopaedics and Traumatology of Zhejiang Province, Hangzhou, Zhejiang 310053, China; ^3^Department of Orthopaedic Surgery, The First Affiliated Hospital of Zhejiang Chinese Medical University, Hangzhou, Zhejiang 310006, China

## Abstract

This study aimed to investigate if autologous bone marrow mesenchymal stem cells (MSCs) could treat osteonecrosis of the femoral head (ONFH) and what the fate and distribution of the cells are in dogs. Twelve Beagle dogs were randomly divided into two groups: MSCs group and SHAM operated group. After three weeks, dogs in MSCs group and SHAM operated group were intra-arterially injected with autologous MSCs and 0.9% normal saline, respectively. Eight weeks after treatment, the necrotic volume of the femoral heads was significantly reduced in MSCs group. Moreover, the trabecular bone volume was increased and the empty lacunae rate was decreased in MSCs group. In addition, the BrdU-positive MSCs were unevenly distributed in femoral heads and various vital organs. But no obvious abnormalities were observed. Furthermore, most of BrdU-positive MSCs in necrotic region expressed osteocalcin in MSCs group and a few expressed peroxisome proliferator-activated receptor-*γ* (PPAR-*γ*). Taken together, these data indicated that intra-arterially infused MSCs could migrate into the necrotic field of femoral heads and differentiate into osteoblasts, thus improving the necrosis of femoral heads. It suggests that intra-arterial infusion of autologous MSCs might be a feasible and relatively safe method for the treatment of femoral head necrosis.

## 1. Introduction

Osteonecrosis of the femoral head (ONFH) is a debilitating disease that frequently affects patients between 20 and 50 years old [1]. It is often motived by insufficient blood supply to trabecular bone and bone cell death in the femoral head for various reasons such as trauma, intemperance, application of hormones, and connective tissue diseases, leading to articular cartilage collapse and subsequent osteoarthritis [2–4]. If untreated timely, it usually leads to the collapse of the femoral head and the total hip replacement is required [5]. As most of those suffering from the disease are young adults, the long-term results of total hip arthroplasty are unpredictable for them [6, 7]. Other treatment options for early stage ONFH include pharmacotherapy, physical therapy, and palliative surgical interventions such as core decompression, rotational osteotomy, and nonvascularized or vascularized bone grafting. Nevertheless, the efficacy of these procedures remains either variable or controversial and palliative surgical interventions have disadvantages of difficult manipulation [2, 8]. It is badly in need of developing noninvasive or less-invasive and effective strategies to protect the femoral head from collapse. Recent studies showed that ONFH is highly related to the decrease or alterations of marrow mesenchymal stem cells (MSCs) or other progenitor cells in proximal femurs [9–13], and it is gradually accepted that ONFH originates from the cellular level [14–16].

MSCs which were well known can differentiate into specialized cells to repair injured tissues, under certain condition as a result of the potential capacity of multidirectional differentiations [17]. Research in recent years has shown a wide application of stem cell transplantation in many ischemic diseases including myocardial infarction, limb ischemia, retinal degeneration, and stroke. Autologous bone marrow transplantation in ONFH patients was first reported by Hernigou et al. [18]. After more clinical studies have been reported, stem cell transplantation had gradually emerged as a promising approach for the treatment of ONFH [19–24]. However, the delivery approaches are still a widely concerned and unsolved problem, and intra-arterial infusion of stem cells as a therapeutic strategy is less studied in the treatment of ONFH. Both the specific mechanism to improve the necrosis of femoral head and the distribution of transplanted MSCs were unclear.

In the present study, we investigated if MSCs could treat ONFH and the fate and distribution of autologous MSCs with intra-arterial infusion in ONFH in dogs.

## 2. Materials and Methods

### 2.1. Animal Models and Groups

Twelve mature male Beagle dogs (10 ± 0.5 kg) were employed for the present study. The study was performed in the Animal Experiment Center of Zhejiang Chinese Medical University and all procedures were carried out in strict accordance with the guidelines for the Care and Use of Laboratory Animals.

The animals were randomly divided into two groups: MSCs group (*n* = 6) and SHAM operated group (*n* = 6). The dog models of ONFH were established by using a liquid nitrogen freezing method similar to what was previously described [25]. All the dogs were intramuscularly anesthetized with ketamine hydrochloride (25 mg/kg, Abbott, USA) and maintained with sodium pentobarbital (5 mg/kg per hour, Abbott, USA). Then a 5 cm long incision was made at the lateral side of the right hip. Via the gap between gluteus medius and gluteus superficialis, the joint capsule was exposed and opened with T-shaped incision. Weight-bearing area of femoral heads was exposed and its surroundings were filled by dry gauze, and 100–150 mL liquid nitrogen was applied to freeze femoral head through a special funnel for three minutes. Instantly, the femoral heads were rewarmed with normal 37°C saline for 3 min. Finally, the wounds were bandaged with sterile dressing and 40,000 UI/kg of penicillin was intramuscularly administered for each dog daily for consecutive 5 days.

### 2.2. Isolation, Culture, and Identification of MSCs

Bone marrow (15 mL) was aspirated from the iliac crest of the dogs using 5 mL syringes and an 18 G bone marrow biopsy needle (Biomedical, USA). The cells were plated in 25 cm^2^ flasks at initial density of 2.0 × 10^5^/cm and incubated in an incubator (Thermo Electron Corporation, USA) at 37°C, relative humidity of approximately 100%, and 5% CO_2_.

MSCs immunophenotypic study was performed by direct staining protocol with conjugated primary monoclonal antibodies. Cell suspensions in third passage were split into five aliquots, a control group stained with an appropriate isotype-matched, nonreactive, fluorochrome-conjugated antibody, and other, respectively, stained with anti-CD45 conjugated with phycoerythrin (CD45-PE, BD Biosciences, San Diego, USA), anti-CD90 conjugated with PE (CD90-PE, BD Biosciences, San Diego, USA), anti-CD11b/c conjugated with phycoerythrin (CD11b/c-PE, eBioscience, San Diego, USA), and anti-CD29 conjugated with phycoerythrin (CD29-PE, eBioscience, San Diego, USA). After addition of antibodies, the cells were incubated for 30 minutes in the dark at 4°C. Detections were conducted with a flow cytometry (FACScan, BD Biosciences, San Diego, USA).

### 2.3. Labeling and Intra-Arterial Infusion of MSCs

To label MSCs, we used 5-bromo-2-deoxyuridine (BrdU, Sigma, USA). BrdU was added to MSCs at a final concentration of 10 *μ*mol/L, when cells were 50–60% confluence at the third passage. After cells reached 90% confluence, cells were harvested and stored on ice until transplanting. Before grafting, a sample of MSCs was collected to assess cell-labeling efficacy.

Three weeks after establishment of ONFH model, intra-arterial infusion was performed on all dogs. The right femoral artery was punctured by the Seldinger technique with a 4.0 F Cobra catheter (Terumo, Japan). One millilitre of MSCs with a cell density of 5 × 10^6^–1 × 10^7^/mL was intra-arterially injected and 0.9% normal saline was used in SHAM operated group. An identical surgical procedure was performed in all dogs except for the different perfusions in each group.

### 2.4. Analysis of Magnetic Resonance Imaging (MRI)

MRI was performed by a Signa Excite 1.5 T imager (GE Medical Systems, Milwaukee WI, USA) at eight weeks after intra-arterial infusion with coronal T1-weighted imaging, T2-weighted imaging and axial T1-weighted imaging, T2-weighted imaging. Images of 3 mm thickness with a 0.5 mm gap were obtained by using a 256 × 192 matrix and four excitations. The volumetric analysis of the osteonecrosis of the femur was determined by measuring the necrotic area (cm^2^) per MRI slide [26, 27]. Then the respective volumes from the respective area multiplied by the thickness of the MRI slide were added to the total volume of the necrosis. In order to assess the validity of the method of measurement, the femoral heads were evaluated independently by three independent investigators.

### 2.5. Measurement of Histology, Immunohistochemistry, and Immunofluorescence

All dogs were sacrificed at eight weeks after intra-arterial infusion. The right femoral heads and vital organs such as heart, lung, liver, spleen, kidney, small bowel, gallbladder, pancreatic, and prostate were harvested.

In order to observe the general architecture and osteonecrosis of the femoral heads, the bone sections (3 *μ*m thickness) were stained with haematoxylin and eosin staining (H&E). The diagnostic criteria for osteonecrosis were defined as diffuse empty lacunae in the trabecular bone or condensed nuclei in the bone cells, accompanied by surrounding bone marrow cell necrosis [28]. The histopathological changes were examined with a light microscope (Zeiss Axio Scope A1, Carl Zeiss Co., Ltd., Jena, Germany) in a blinded manner by three independent investigators. Using 100x magnification, 5 fields were selected randomly and at least 50 lacunae were counted in each field. The ratio of empty lacuna number to the total lacuna count defined the percentage of empty lacunae. The trabecular bone volume was measured by Image-Pro Plus 6.0 (Media Cybernetics, USA) and the percentage of trabecular bone volume/total volume of tissue was calculated.

To investigate the distribution of grafted cells, the bone and organ sections were also prepared for BrdU assay. The sections were incubated with 1 : 300 mouse anti-BrdU (Sigma, USA) overnight at 4°C. After rinsing, sections were incubated with 1 : 200 secondary biotinylated goat anti-mouse antibody (Vector, USA) for 30 minutes at room temperature. Following being incubated with 1 : 250 streptavidin-peroxidase (Pierce, USA) for 30 minutes at room temperature, peroxidase activity was revealed by diaminobenzidine (Histofine DAB-3S kit, Nichirei, Japan), and sections were counterstained with hematoxylin. The tissues sections were examined under a light microscope (Zeiss Axio Scope A1, Carl Zeiss Co., Ltd., Jena, Germany). We quantified BrdU-positive cells in four randomly selected fields (one field = 25 × 10^−8^ m^2^) from each of the three different tissue sections by Image-Pro Plus 6.0 (Media Cybernetics, USA).

To study the differentiation of grafted cells, double-label immunofluorescence was used for bone sections. After blocking nonspecific binding sites with 1 : 20 normal goat serum (Vector, USA) for 30 minutes, the sections for osteogenic differentiation were incubated with primary antibody: anti-BrdU antibody (ab6326, 1 : 40, Abcam, UK) and anti-osteocalcin antibody (ab13420, 10 *μ*g/mL, Abcam, UK), overnight at 4°C. The sections for adipogenic differentiation were incubated with primary antibody: BrdU (Bu20a) Mouse mAb (#5292, 1 : 200, Cell Signaling Technology, USA) and anti-PPAR gamma antibody (ab19481, 4 *μ*g/mL, Abcam, UK), overnight at 4°C. After that, the specimens were, respectively, incubated in corresponding fluorochrome-conjugated secondary antibody mixture for 1 h at room temperature in the dark and mounted coverslip after DAPI (C0060, 1 : 1000, Solarbio, China) staining. Specimens were examined by a light microscope (Zeiss Axio Scope A1, Carl Zeiss Co., Ltd., Jena, Germany). Evaluations were performed by randomly taking five higher power fields under 400X magnification and the percentage of BrdU^+^/osteocalcin^+^ cells or BrdU^+^/PPAR-*γ* ^+^ cells over total BrdU^+^ cells was calculated and compared.

### 2.6. Statistical Analysis

Data were presented as mean ± SD. Paired *t*-test was used to compare the difference between the MSCs group and the SHAM operated group. The statistical tests were performed by using the software SPSS V17.0. *p* < 0.05 was considered statistically significant.

## 3. Results

### 3.1. Evaluation of MSCs and BrdU-Labeling Efficacy

**Figure 1:**
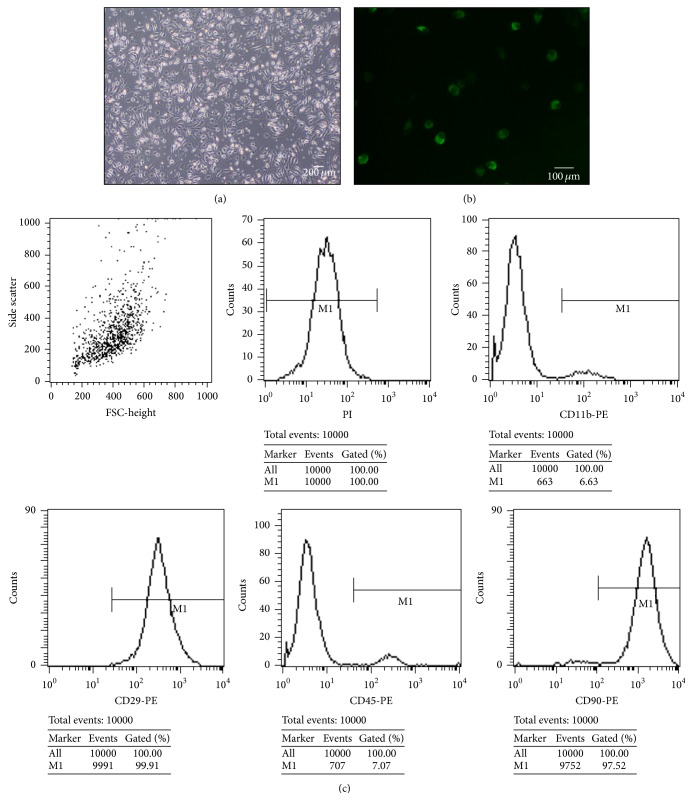
Evaluation of MSCs and BrdU-labeling efficacy. Primary cultured MSCs exhibit typical elongated, fibroblast-like morphology or large, flattened shape (a).* Scale bar 200 μm*. MSCs in third passage were positive for BrdU, as immunofluorescence stained with BrdU (b).* Scale bar 100 μm*. Flow cytometry analysis of the surface markers of bone marrow mononuclear cells: CD29 (99.91%), CD90 (97.52%), CD11b (6.63%), and CD45 (7.07%) (c).

MSCs were successfully cultured and identified by surface markers: CD45, CD90, CD11b/c, and CD29. The evaluation was observed by a phase contrast microscope (Olympus, Japan) 24 hours after MSCs were seeded, when part of the round mononuclear cells was adherent. Four days after inoculation, small colonies of the adherent cells with typical fibroblast-shaped morphology were obtained. These primary cells reached monolayer confluence, after planting for 10–14 days, when passaged for the first time. The primary cultured MSCs exhibited typical elongated, fibroblast-like morphology or large, flattened shape (Figure 1(a)). And flow cytometric analysis showed that they were strongly positive for CD29 (99.91%) and CD90 (97.52%), but negative for CD11b/c (6.63%) and CD45 (7.07%) (Figure 1(c)). Immunofluorescence staining of MSCs in third passage showed that they were positive for BrdU which meant an available cell-labeling efficacy (Figure 1(b)).

### 3.2. Regression of a Necrotic Lesion after Intra-Arterial Infusion on MRI

**Figure 2:**
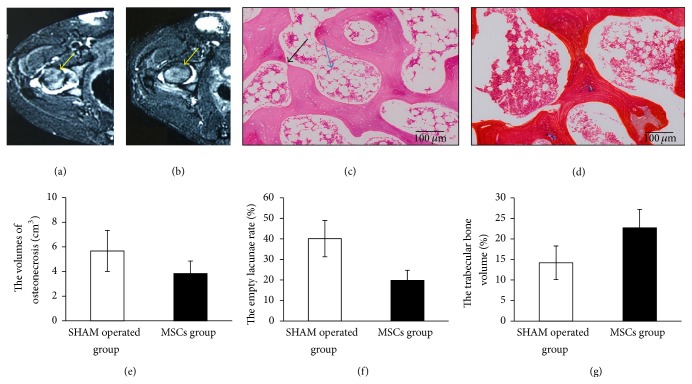
The signs of ONFH in imaging and histopathology at 8th week after treatment. Axial T2-weighted MRI in SHAM operated group showed intra-articular effusion, irregular femoral head margin, and flattened and smaller femoral head (a). Axial T2-weighted MRI in MCSs group showed marked reduction in lesion size, profile rules, and comparatively smooth edge (b). The result from the volumetric analysis showed that the necrotic volume of femoral heads in MSCs group was smaller than that in SHAM operated group (*p* < 0.05) (e). Histological findings show disordered trabeculae, incomplete, significant degeneration, and necrosis with slight fractures in SHAM operated group (c) after eight-week treatment whereas the morphological structure of the MSCs group relatively improved (d).* Scale bar 100 μm*. Black arrow shows the slight fracture and blue arrow shows the fat cell hypertrophy. Comparison of the empty lacunae rate between MSCs group and SHAM operated group, the empty lacunae rate in SHAM operated group was significantly higher than that of MSCs group after eight-week treatment (*p* < 0.05) (f). The percentage of trabecular bone volume in MSCs group was significantly higher than that in SHAM operated group after eight-week treatment (*p* < 0.05) (g).

MRI was performed to determine conditions of ONFH after eight weeks of treatments. Dogs in SHAM operated group showed a considerable area of low signal intensity on T1W, slightly high signal on T2W, intra-articular effusion, irregular femoral head margin, and flattened and smaller femoral head (Figure 2(a)), while dogs in MSCs group showed sporadic low signal intensity on T1W, but no obvious abnormal signal intensity on T2W and STIR, profile rules, and comparatively smooth edge (Figure 2(b)).

The result from the volumetric analysis showed that the necrotic volume of femoral heads in SHAM operated group is 5.67 ± 1.67 cm^3^; in contrast the necrosis volume in MSCs group decreased to 3.84 ± 1.01 cm^3^ (*p* < 0.05) (Figure 2(e)).

### 3.3. Morphological Changes in Necrotic Field of Femoral Head

At eight weeks after treatment, the SHAM operated group showed incomplete periosteum, severely thinned and disordered trabeculae, massive empty bone lacunae, pyknotic nuclei of osteocytes, decreased haemopoietic cells, and large amounts of fat cells by using HE staining (Figure 2(c)), while partly trabeculae structures were recovered in MSCs group. And at the same time the normal bone cells were presented and the number of fat cells in bone marrow was decreased in MSCs group (Figure 2(d)). Moreover, the empty lacunae rate was significantly lower in MSCs group (*p* < 0.05) (Figure 2(f)) and the percentage of trabecular bone volume was significantly higher (*p* < 0.05) (Figure 2(g)). It may indicate that autologous MSCs with intra-arterial infusion could obviously improve trabecular bone shape, decrease empty lacunae, and reduce fat cell numbers during the early ONFH.

### 3.4. Fate and Distribution of MSCs with Intra-Arterial Infusion* In Vivo*

**Figure 3:**
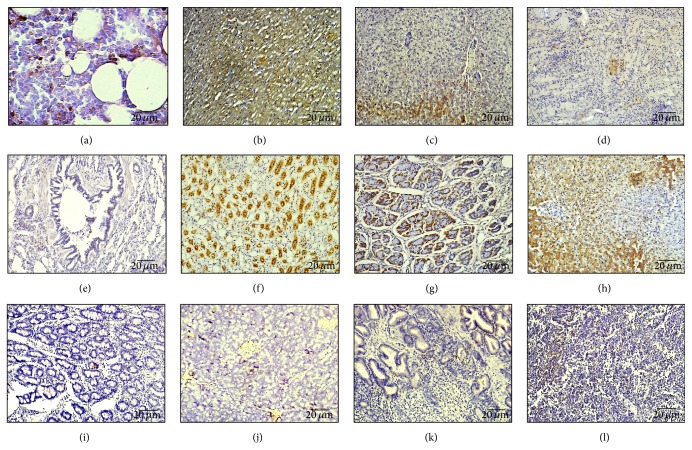
The distribution of MSCs with intra-arterial infusion in femoral head and vital organs. Immunohistochemical staining of BrdU-positive MSCs in different tissues at eight weeks after transplantation was observed under a light microscope. (a) Femoral head. (b) Heart; (c) liver; (d) spleen; (e) lung; (f) kidney; (g) stomach; (h) gallbladder; (i) small bowel; (j) pancreas; (k) prostate; (l) testicle.* Scale bar 20 μm*. Particularly, BrdU strongly positive MSCs were significantly located in the tissues of the kidney, gallbladder, and liver.

Eight weeks after intra-arterial infusion, BrdU-positive cells were unevenly observed in vital organs by using immunohistochemistry, which included right femoral head, heart, lung, liver, spleen, kidney, gallbladder, small bowel, pancreas, prostate, and testicle. In the femoral head, BrdU-positive MSCs were accounted for 21.58 ± 2.06%. The more BrdU-positive cells are shown in heart (64.25 ± 4.79%), gallbladder (60.26 ± 3.88%), kidney (46.36 ± 5.14%), liver (32.50 ± 3.70%), and stomach (35.52 ± 4.51%) (Table 1). In morphology, the positive cells in kidney are formed primarily by renal tubular epithelial cells, in gallbladder are formed primarily by gallbladder epithelial cells, in liver are formed primarily by hepatocytes, and in heart are formed primarily by cardiomyocytes (Figures 3(a)–3(l)). However, the presence in intestine (6.08 ± 1.57%), spleen (4.21 ± 1.44%), and lung (3.54 ± 1.23%) is only a small amount of BrdU-positive cells (Table 1).

**Figure 4:**
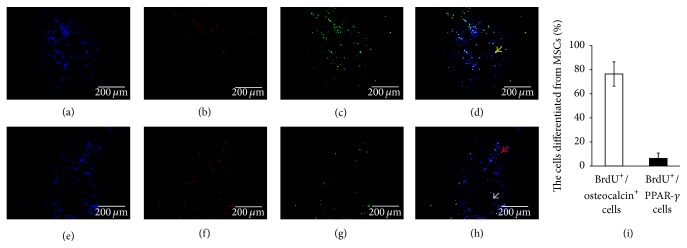
Osteogenic and adipogenic differentiation of MSCs with intra-arterial infusion in necrotic field of femoral head. BrdU (green) and osteocalcin (red) were analyzed by double-immunofluorescent analysis in the necrotic region of MSC-transplanted femoral heads. (a) DAPI (blue); (b) BrdU (red); (c) osteocalcin (green); (d) an overlay of (a), (b), and (c); (e) DAPI (blue); (f) BrdU (red); (g) PPAR-*γ* (green); (h) an overlay of (e), (f), and (g). Most of the BrdU-positive MSCs in the necrotic region of MSC-transplanted femoral heads costained together with osteocalcin but a few cells costained together with PPAR-*γ*. Yellow arrow shows osteoblasts differentiated from MSCs, red arrow shows adipocytes differentiated from MSCs, and grey arrow shows BrdU-positive cells which did not differentiate to lipocytes.* Scale bar 200 μm*. BrdU^+^/osteocalcin^+^ cells in MSCs group (76.42 ± 10.14%) were significantly higher than BrdU^+^/PPAR-*γ* ^+^ cells over total BrdU^+^ cells (6.36 ± 4.41%) after eight-week treatment (*p* < 0.05) (i).

The double-staining of the femoral head sections demonstrated that no BrdU-positive cells were observed in SHAM operated group at eight weeks and the osteocalcin and PPAR-*γ* expressed in the necrotic region of femoral heads were shown in Supplemental Figure  1 in Supplementary Material available online at http://dx.doi.org/10.1155/2016/8616143. Most of the BrdU-positive MSCs (76.42 ± 10.14%) in the necrotic region of MSC-transplanted femoral heads expressed osteocalcin (Figures 4(a), 4(b), 4(c), 4(d), and 4(i)). In addition, a few cells (6.36 ± 4.41%) also expressed PPAR-*γ* (Figures 4(e), 4(f), 4(g), 4(h), and 4(i)).

**Figure 5:**
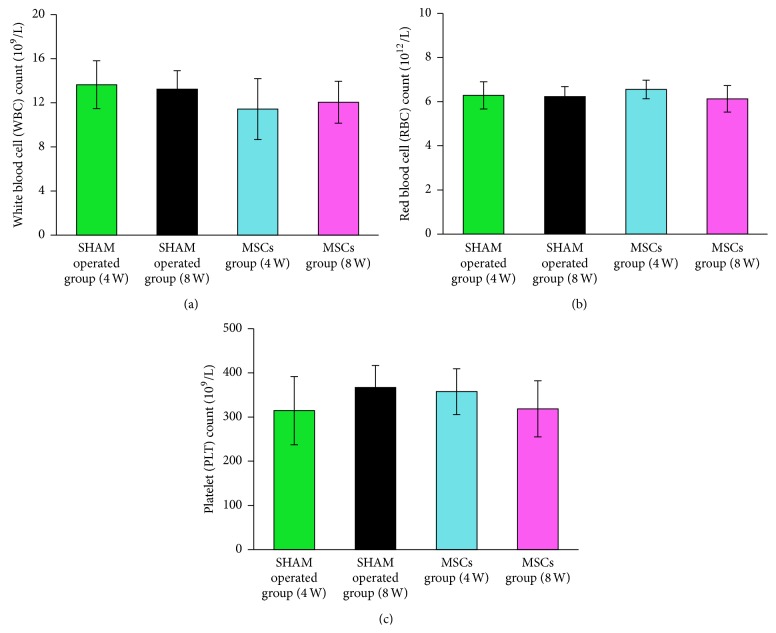
The difference of white blood cell (WBC) counts, red blood cell (RBC) counts, and platelet (PLT) counts between the two groups at 4th and 8th week after treatment. At 4th and 8th week after treatment, the white blood cell (WBC) counts, red blood cell (RBC) counts, and platelet (PLT) counts between MSCs group and SHAM operated group showed no significant difference (*p* > 0.05) (a, b, c).

Moreover, to evaluate the potential safety of autologous MSCs with intra-arterial infusion, we observed the general condition. During the whole experiment, all animals survived. At 4th and 8th week after treatment, no obvious abnormalities were observed between MSCs group and SHAM operated group in the mental condition, body temperature, and body weight. Furthermore, routine blood tests showed no significant difference, including WBC, RBC, and PLT (Figures 5(a), 5(b), and 5(c)).

## 4. Discussion

Nontraumatic ONFH is an impaired skeletal disorder that usually leads to collapse of the femoral head. And the potential less-invasive application of stromal cells has become one of the treatments of ONFH [19, 22–24]. A double-blind nonrandomized study has indicated that delivering autologous bone marrow mononuclear cells to patients with early stages of ONFH brought about a profound reduction in pain and in joint symptoms and partly prevented the occurrence of fractural stages [19]. There are three methods to transplant MSCs up till now, including local injection [29–32], intravenous delivery [33–37], and targeted intra-arterial injection [38, 39]. And the core decompression with autologous bone marrow cell implantation is the most common strategy for the cell therapy of ONFH [40]. But the operation process is relatively invasive and complex. Although the intravenous delivery, as the major route of administration of MSC, is simple and convenient, MSCs delivered systemically are trapped into various tissues by using this method, especially the lungs [41–43]. And the transfer efficiency turns relatively low. It has been proved that the delivery of MSCs via the targeted artery can significantly increase the cells number of homing into the injured tissue and improve function compared with injection via the intravenous route [39, 44]. Some researchers also conceded that ONFH was a coronary disease of the hip [45]. The blood supply of both the femoral head and the heart is dependent on nutrient vessels that are torturous with limited anastomoses supply [45]. The medial circumflex femoral artery is the main nutrient vessel of the femoral head, just like coronary artery in the heart. However, few evidences on fate and distribution of stem cells intra-arterial infusion were found in the treatment of ONFH. In the present study, we investigated the fate and distribution of autologous MSCs with intra-arterial infusion in ONFH of dogs to assess the feasibility and safety.

MSCs are multipotent stem cells which can differentiate into various connective tissue lineages. According to the International Society for Cell Therapy [46], human MSCs were defined as plastic adhering cultured cells in standard culture conditions that express CD105, CD73, and CD90 and lack expression of CD45, CD34, CD14, or CD11b, CD79 alpha or CD19 and HLA-DR surface molecules, while maintaining the capacity for differentiating into osteoblasts, adipocytes, and chondrocytes* in vitro*. Our data shows that MSCs strongly expressed CD29 (99.91%) and CD90 (97.52%) but negatively expressed CD11b/c (6.63%) and CD45 (7.07%) (Figure 1(c)); it is reasonable to assume that MSCs obtained in present study met the criteria set by the International Society for Cell Therapy [46]. BrdU was used to label stem cell in our study. BrdU not only can selectively incorporate into DNA at the S phase of the cell cycle (DNA synthesis phase) but also does not affect cell growth, proliferation, and differentiation. Furthermore it can be tracked by immunohistochemistry and immunofluorescence.

Based on the MRI results, the femoral heads in MSCs group showed sporadic low signal intensity on T1W, no obvious abnormal signal intensity on T2W and STIR, profile rules, and comparatively smooth edge after eight weeks of treatments (Figures 2(a) and 2(b)). The rate of radiological progression on necrotic lesion in MSCs group was statistically lower than that in SHAM operated group. Moreover, the results from the volumetric analysis showed the necrotic volume of femoral heads in MSCs group decreased to a degree (Figure 2(e)). Taken together, these results indicate that autologous MSCs with intra-arterial infusion could obviously improve trabecular bone shape, decrease empty lacunae, and reduce fat cell numbers during the early ONFH.

Since the effect of MSCs with intra-arterial infusion in ONFH is comparatively remarkable, the specific mechanism of intra-arterial infusion of MSCs is getting more and more attention. Previous studies on the immunogenicity of MSCs reveal that the immunological rejection was reduced in allogeneic transplantation by inhibiting cell proliferation [47]; to avoid the immunological rejection, the autologous MSCs were employed in present study. The immunohistochemical data showed that partial BrdU-positive MSCs could migrate into the necrotic field of femoral head (21.58 ± 2.06%). And BrdU-positive MSCs were also trapped into various tissues, especially heart (64.25 ± 4.79%), gallbladder (60.26 ± 3.88%), kidney (46.36 ± 5.14%), liver (32.50 ± 3.70%), and stomach (35.52 ± 4.51%) (Table 1), while they could differentiate into various connective tissue lineages in morphology similar to their own cells in corresponding organ. The blood flow of liver and kidney is more abundant which might even explain why more BrdU-positive cells appear in the tissues like the liver and kidney. However, as to the gallbladder, it seems to be not adequate to account for it.

As we know, MSCs have the potential to differentiate into osteoblasts, adipocytes, and so on. In order to investigate the fate of MSCs transplanted by intra-arterial infusion in ONFH dog's models, we performed a double-staining experiment to confirm if they were differentiated into other lineages or not. As a symbol of mature osteoblasts, osteocalcin is secreted solely by osteoblasts and thought to play a role in the body's metabolic regulation and bone formation. The immunofluorescence pictures demonstrated that the most BrdU-positive MSCs (76.42 ± 10.14%) in the necrotic region of femoral heads expressed osteocalcin in MSCs group (Figures 4(a), 4(b), 4(c), and 4(d)). It indicated that the transplanted MSCs appearing in the necrotic region of femoral heads could differentiate into osteoblasts, similar to the previous study [48]. In addition, we also checked PPAR-*γ* in MSCs, as it is a crucial role in regulating adipocytes formation. It can trigger the adipogenesis/osteoblastogenesis switch [49]. We found that only a few adipocytes derived from the transplanted MSCs (6.36 ± 4.41%) (Figures 4(e), 4(f), 4(g), 4(h), and 4(i)). It may explain that MSCs with intra-arterial infusion are inclined to commit to repair and regeneration in the condition of bone necrosis. The inflammatory factors were considered to potently enhance the osteogenic capacity of MSCs which may be propitious to bone regeneration [50]. Therefore, we considered that intra-arterial infused autologous MSCs in ONFH could migrate into the necrotic field of femoral head and multiply differentiate into osteoblasts. And it may be an important mechanism to improve above signs of ONFH in imaging and histopathology.

More importantly, no obvious abnormalities on the mental condition, body temperature, and body weight were observed in our study and no significant difference on routine blood tests appeared, including WBC, RBC, and PLT (Figures 5(a), 5(b), and 5(c)) between MSCs group and SHAM operated group. Similar results on safety have been reported in previous study by Li et al. They injected allogeneic MSCs into rabbits and found that no local or systematic manifestations of toxic reactions and graft versus host disease were observed during and after transplantation [51]. Based on the above results, we believed that this approach is relatively safe.

Consequently, we considered autologous bone marrow mesenchymal stem cells with intra-arterial infusion might be a feasible and relatively safe avenue for the treatment of ONFH. However, the problem of whether the differences existed between the normal population of osteoblasts and the osteoblasts derived from MSCs is truly worthy of consideration. But we regret that this problem had been ignored and its left side (uninjured side) was not reserved during the collection of samples. In addition, the long-term safety of autologous MSCs with intra-arterial infusion needs further evaluation.

## Supplementary Material

The immunofluorescent analysis in SHAM operated group showed the osteocalcin could express in the necrotic region of femoral heads (Supplemental Figures 1(a), 1(b), 1(c)). Besides, more PPAR-γ expressed in SHAM operated group than that in MSCs group (Supplemental Figures 1(d), 1(e), 1(f)).

## Figures and Tables

**Table 1:** The ratio of BrdU-positive cell over total cell number in different tissues at eight weeks after intra-arterial infusion of MSCs.

Tissues	The ratio of BrdU-positive cell over total cell number (%)
Femoral head	21.58 ± 2.06
Heart	64.25 ± 4.79
Liver	32.50 ± 3.70
Spleen	4.21 ± 1.44
Lung	3.54 ± 1.23
Kidney	46.36 ± 5.14
Stomach	35.52 ± 4.51
Gallbladder	60.26 ± 3.88
Intestine	6.08 ± 1.57
Pancreas	10.25 ± 1.50
Testicle	15.56 ± 4.74
Prostate	10.18 ± 2.10
