# Supplementary material for: The Fate and Distribution of Autologous Bone Marrow Mesenchymal Stem Cells with Intra-Arterial Infusion in Osteonecrosis of the Femoral Head in Dogs
Source: Stem Cells Int. 2015 Dec 8;2016:8616143. doi: 10.1155/2016/8616143 (PMC4686726; doi:10.1155/2016/8616143)
Supplement: Supplementary file 1 — The immunofluorescent analysis in SHAM operated group showed the osteocalcin could express in the necrotic region of femoral heads (Supplemental Figures 1(a), 1(b), 1(c)). Besides, more PPAR-γ expressed in SHAM operated group than that in MSCs group (Supplemental Figures 1(d), 1(e), 1(f)). [file 8616143.f1.docx]

**Supplemental Information**

**The fate and distribution of autologous bone marrow mesenchymal stem cells with intra-arterial infusion in osteonecrosis of the femoral head in dogs**

Hongting Jin^1,2¶^, Taotao Xu^1,2¶^, Qiqing Chen^1,2^, Chengliang Wu^1,2^, Pinger Wang^1,2^, Qiang Mao^3^, Shanxing Zhang^1,2^, Jiayi Shen^1,2^, Peijian Tong^3*^

^1^Zhejiang Chinese Medical University, Hangzhou 310053, Zhejiang Province, China

^2^Institute of Orthopaedics and Traumatology of Zhejiang Province, Hangzhou 310053, Zhejiang Province, China

^3^Department of Orthopaedic Surgery, the First Affiliated Hospital of Zhejiang Chinese Medical University, Hangzhou 310006, Zhejiang Province, China

Hongting Jin, E-mail: hongtingjin@163.com

Taotao Xu, E-mail: nacle1990829@163.com

Qiqing Chen, E-mail: zy.chenqiqing@163.com

Chengliang Wu, E-mail: wcl@zcmu.edu.cn

Pinger Wang, E-mail: apple63209321@126.com

Qiang Mao, E-mail: peijiantongzy@126.com

Shanxing Zhang, E-mail: 15700050235@163.com

Jiayi Shen, E-mail: 15158190224@163.com

*****Corresponding author

Peijian Tong, M.D.

The First Affiliated Hospital of Zhejiang Chinese Medical University,

You Dian Road NO.54, Hangzhou 310006

Zhejiang Province

People’s Republic of China

Tel: 011-86 571-86613684; Fax: 011-86 571-86613684

Email: [peijiantongzjtcm@163.com]

¶These authors contributed equally to this work.

**Supplemental Figure 1. The immunofluorescent analysis of osteocalcin and PPAR-γ in the necrotic region of femoral heads in SHAM operated group.**


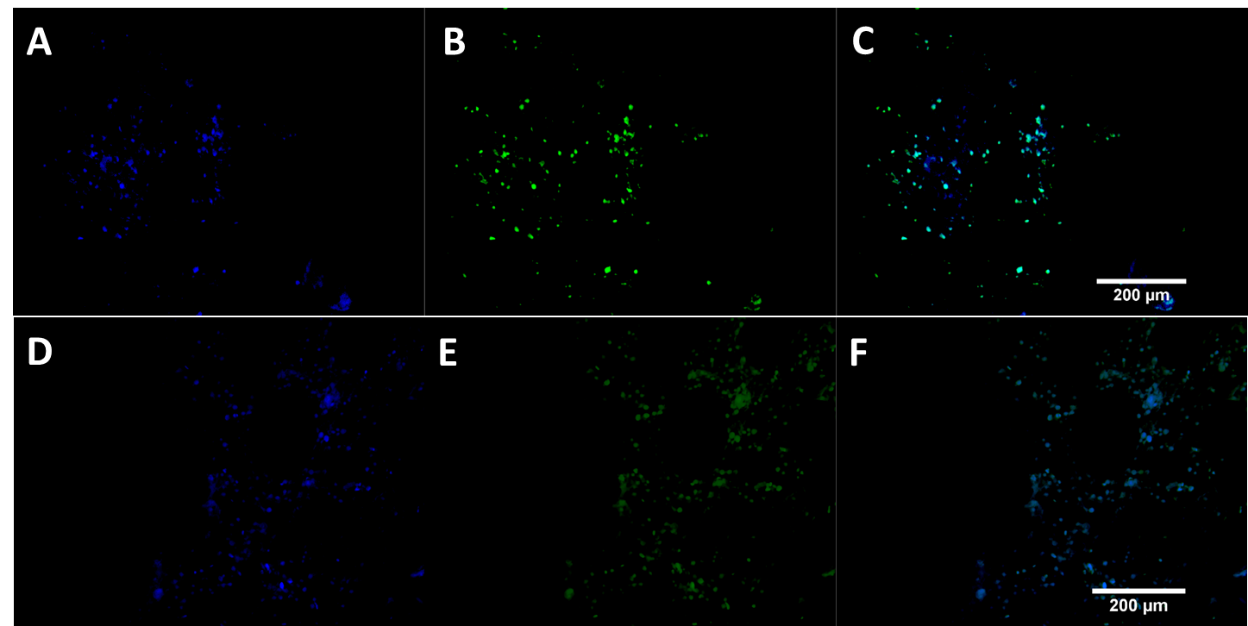


BrdU (green) and osteocalcin (red) were analysed by immunofluorescent analysis in the necrotic region of femoral heads in SHAM operated group. A: DAPI(blue); B: osteocalcin(green); C: an overlay of A and B; D: DAPI(blue); E: PPAR-γ (green); F: an overlay of D and E. *Scale bar 200 μm.*
